# Supplementary material for: Biochemical Fractionation of Human α-Synuclein in a Drosophila Model of Synucleinopathies
Source: Int J Mol Sci. 2024 Mar 25;25(7):3643. doi: 10.3390/ijms25073643 (PMC11011978; doi:10.3390/ijms25073643)
Supplement: Supplementary file 1 [file ijms-25-03643-s001.zip › ijms-2906715-supplementary.pdf]

International Journal of *Molecular Sciences*

MDPI

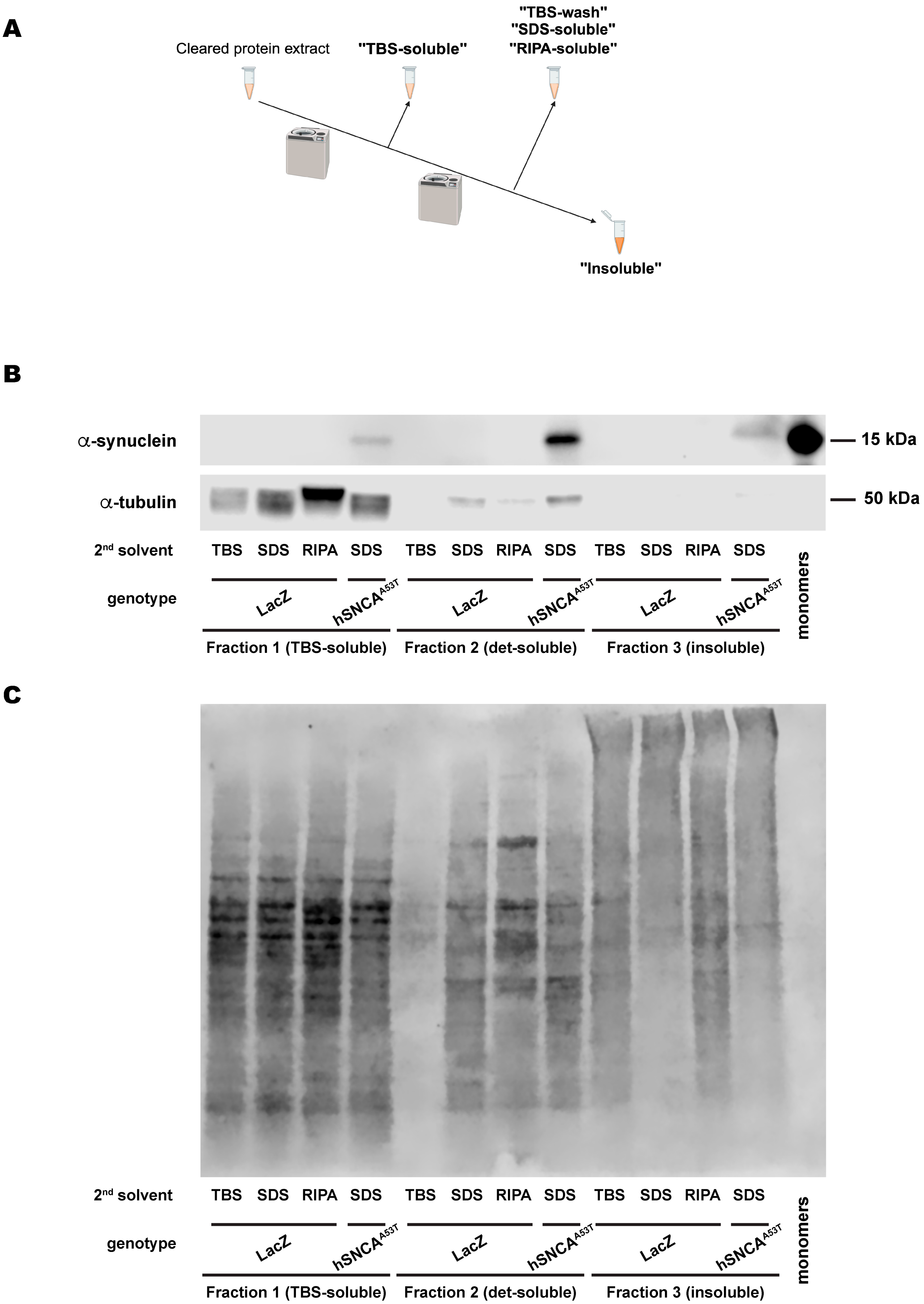

Figure S1. (Related to figure 2). Lack of antibody reactivity in genetic control flies. (A) Schematic representation of the sequential fractionation protocol and the extraction buffers employed in this experiment. **(B)** Representative western blot of head lysates from flies expressing *LacZ* or *hSNCA*^{A53T} in dopaminergic neurons. Fly heads are fractionated using a 3-step protocol in which the second fraction uses a variable detergent solvent, TBS, SDS or RIPA buffer. The first fraction (TBS-soluble) is loaded in lanes 1-4, second fraction (TBS-wash, SDS-soluble or RIPA-soluble) is loaded in lanes 5-8, and the third fraction (insoluble) is loaded in lanes 9-12, while 2ng of purified recombinant human α-synuclein monomers (monomer) are loaded in lane 13 as positive control. Protein lysates are extracted from flies expressing *LacZ* in dopaminergic neurons (*w; +/+; TH-Gal4/ UAS-LacZ*, lanes 1-3, 5-7, 9-11) and flies expressing *hSNCA*^{A53T} (*w; +/+; TH-Gal4/ UAS-hSNCA*^{A53T}, lanes 4, 8, 12). The fractions are probed for α-synuclein (4B12, top panel) and α-tubulin (T6074, bottom panel). Note that control flies (*w; +/+; TH-Gal4/ UAS-LacZ*) do not show any reactivity to the α-synuclein antibody regardless of the fractionation protocol. **(C)** Total protein staining with Revert Total Protein Stain of the membrane employed in this experiment.

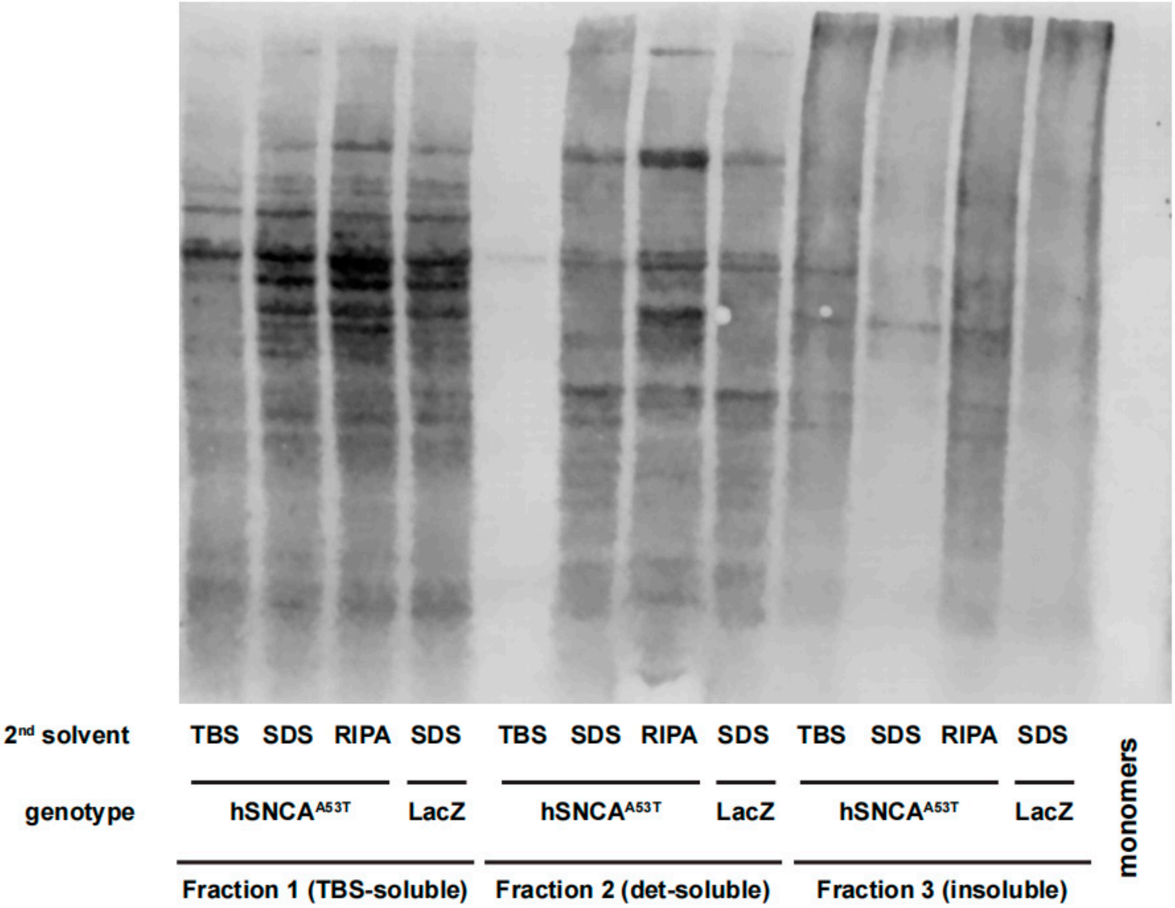

Figure S2. (Related to figure 2). Total protein staining with Revert Total Protein Stain of the membrane employed for the experiment in figure 2.

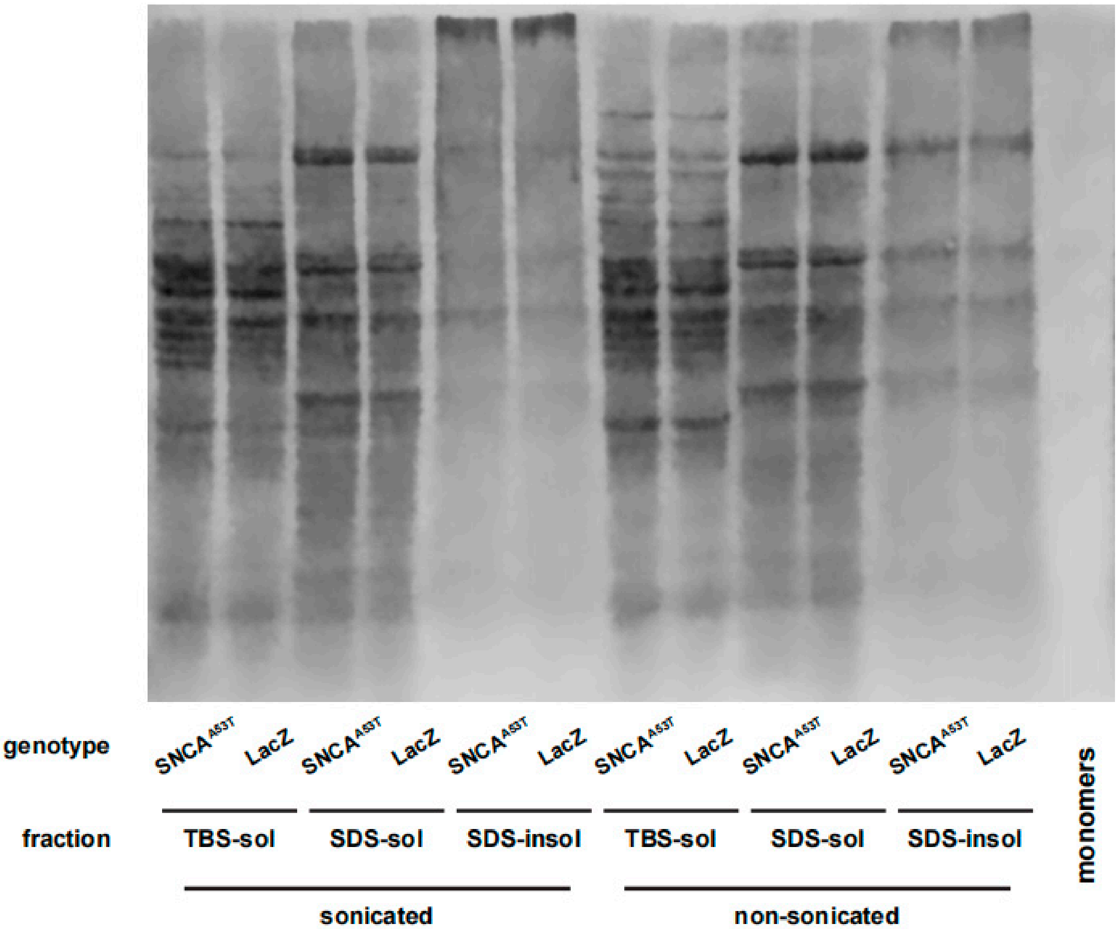

Figure S3. (Related to figure 3). Total protein staining with Revert Total Protein Stain of the membrane employed for the experiment in figure 3.

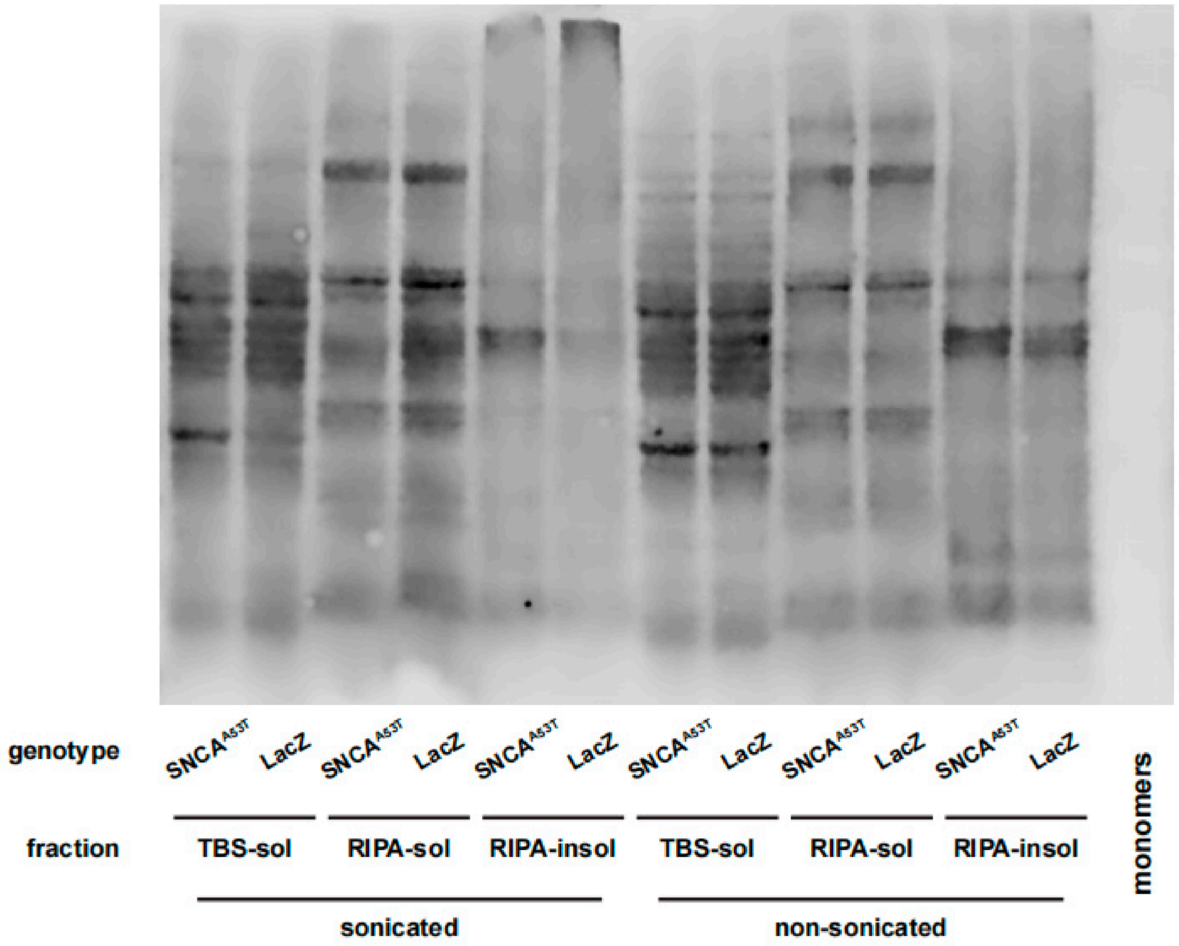

Figure S4. (Related to figure 4). Total protein staining with Revert Total Protein Stain of the membrane employed for the experiment in figure 4.

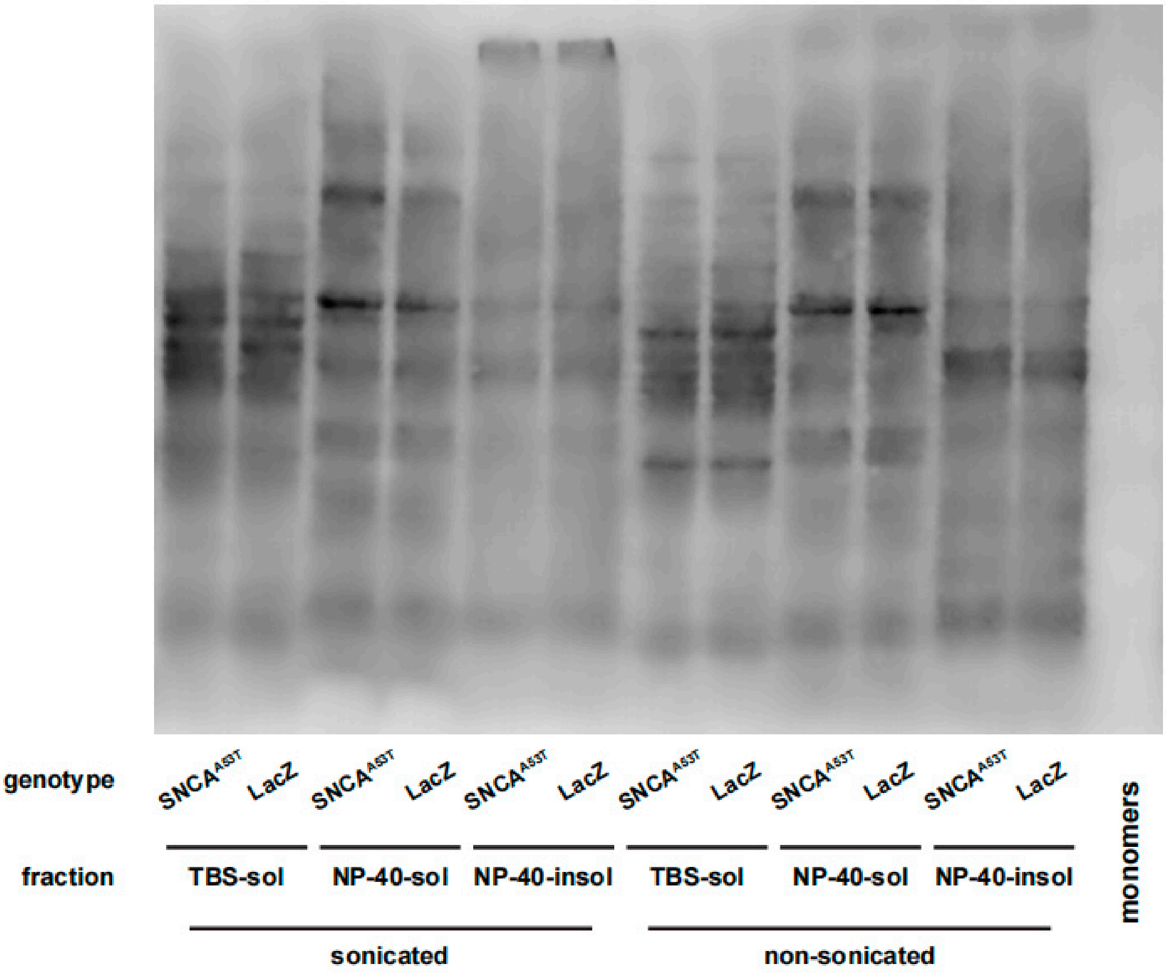

Figure S5. (Related to figure 5). Total protein staining with Revert Total Protein Stain of the membrane employed for the experiment in figure 5.

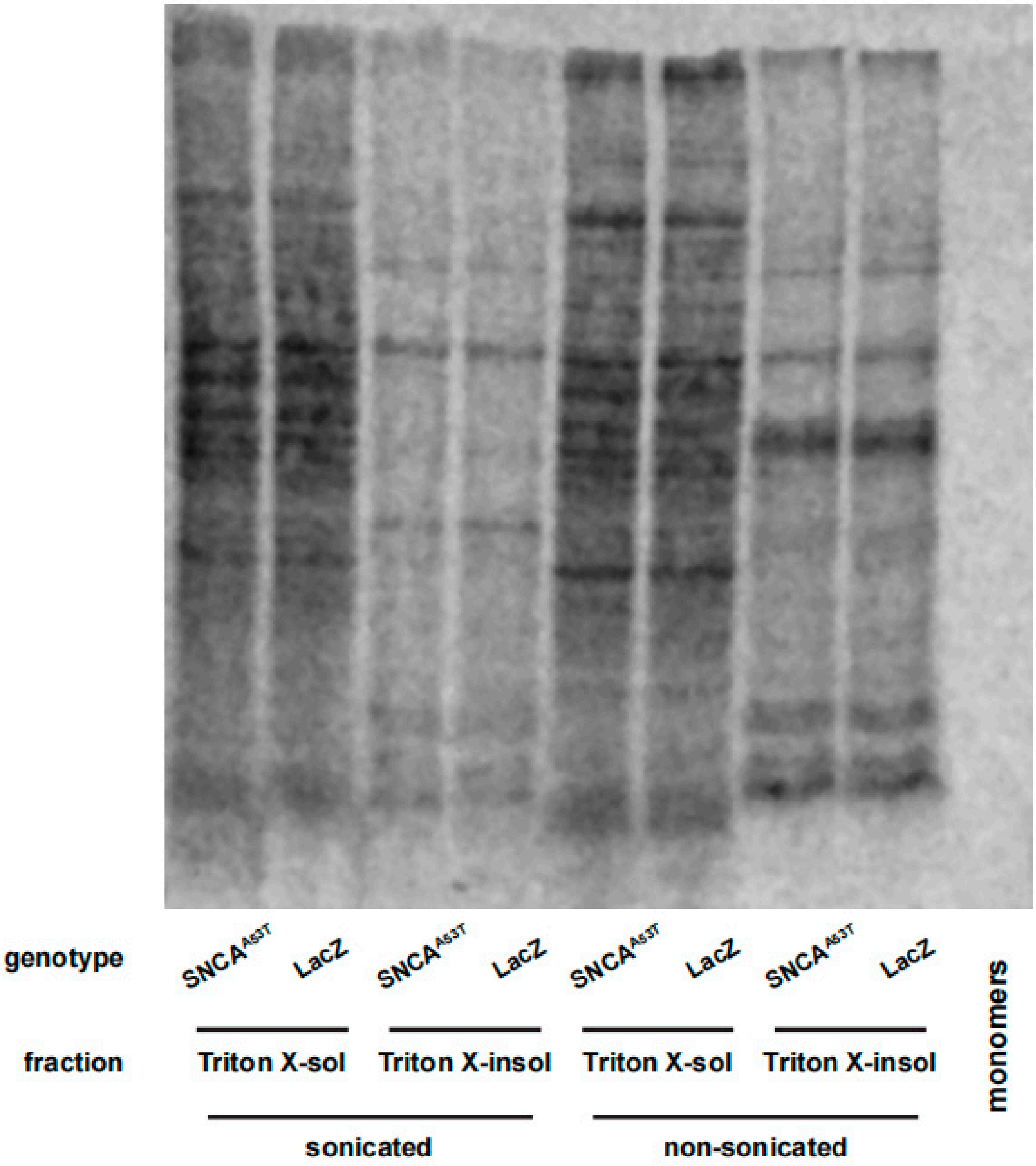

Figure S6. (Related to figure 6). Total protein staining with Revert Total Protein Stain of the membrane employed for the experiment in figure 6.

Table S1. Comparison of α-synuclein enrichment in different fractions. The data show differences in mean values ± SD of normalized α-synuclein signal detected in each biochemical fraction from *TH-Gal4/UAS-hSNCA*^{A53T} flies (sonicated and non-sonicated data were grouped for this analysis), and p-values from *post-hoc* Tukey's multiple comparisons assessing the significance of the differences between the compared groups. Normalization was performed in reference to total protein staining. Signal values are in arbitrary units (a.u.).

**Fractionation Sequence**	**Fractions compared** (fraction A – fraction B)	**Difference** (fraction A – fraction B)	**p-value**
**TBS>SDS>insol**	TBS - SDS	-1.119 ± 0.672	0.0120
	TBS - Insol	-1.059 ± 0.672	0.0176
	SDS - Insol	0.060 ± 0.888	0.9849
**TBS>RIPA>insol**	TBS - RIPA	-3.068 ± 1.504	0.0002
	TBS - Insol	0.429 ± 0.286	0.7676
	RIPA - Insol	3.496 ± 1.479	<0.0001
**TBS>NP-40>insol**	TBS - NP-40	-1.019 ± 0.602	0.0071
	TBS - Insol	0.730 ± 0.090	0.0527
	NP-40 - Insol	1.749 ± 0.596	<0.0001
**Triton>insol**	TritonX - Insol	1.834 ± 0.235	<0.0001

Table S2. Comparison of α-tubulin enrichment in different fractions. The data show differences in mean values ± SD of normalized α-tubulin signal detected in each biochemical fraction from *TH-Gal4/UAS-hSNCA*^{A53T} flies (sonicated and non-sonicated data were grouped for this analysis), and p-values from *post-hoc* Tukey's multiple comparisons assessing the significance of the differences between the compared groups. Normalization was performed in reference to total protein staining. Signal values are in arbitrary units (a.u.).

**Fractionation Sequence**	**Fractions compared** (fraction A – fraction B)	**Difference** (fraction A – fraction B)	**p-value**
**TBS>SDS>insol**	TBS - SDS	2.209 ± 0.537	<0.0001
	TBS - Insol	2.284 ± 0.523	<0.0001
	SDS - Insol	0.075 ± 0.236	0.9418
**TBS>RIPA>insol**	TBS - RIPA	2.081 ± 0.433	<0.0001
	TBS - Insol	2.144 ± 0.431	<0.0001
	RIPA - Insol	0.063 ± 0.088	0.9493
**TBS>NP-40>insol**	TBS - NP-40	3.338 ± 1.203	0.0001
	TBS - Insol	3.271 ± 1.221	0.0001
	NP-40 - Insol	-0.067 ± 0.240	0.9925
**Triton>insol**	TritonX - Insol	2.173 ± 0.375	<0.0001
